# Concomitant of Pulmonary Hydatid Cyst and Aspergilloma: A Rare Coinfection

**DOI:** 10.1155/2020/6650478

**Published:** 2020-12-12

**Authors:** Zahra Zareshahrabadi, Bahador Sarkari, Nadereh Shamsolvaezin, Bizhan Ziaian, Alireza Tootoonchi, Reza Shahriarirad, Kamiar Zomorodian

**Affiliations:** ^1^Department of Parasitology and Mycology, School of Medicine, Shiraz University of Medical Sciences, Shiraz, Iran; ^2^Kowsar Hospitals, Shiraz, Iran; ^3^Department of Thoracic Surgery, Shiraz University of Medical Sciences, Shiraz, Iran; ^4^Thoracic and Vascular Surgery Research Center, Shiraz University of Medical Sciences, Shiraz, Iran; ^5^Basic Sciences in Infectious Diseases Research Center, Shiraz University of Medical Sciences, Shiraz, Iran

## Abstract

The coexistence of cystic echinococcosis (CE) and aspergilloma is rather uncommon. *Aspergillus* species, saprophytic fungi, can colonize pulmonary cavities that are caused by tuberculosis, sarcoidosis, and CE. Infection by *Aspergillus* is often occurring in immunosuppressed patients. However, coinfection of aspergilloma with pulmonary hydatid cyst is very unusual, especially in an immunocompetent patient with unruptured cyst. Herein, we report a case of lung hydatid cyst coinfected with *Aspergillus* in a 42-year-old Iranian man from Southern Iran. Chest X-ray and computed tomography (CT) scan showed a circumscribed cystic lesion in the superior and inferior segment of the lower lobes of right and left lungs that suggests hydatid cyst. Radical surgery (lobectomy) was performed for the patient. Histopathological evaluation reconfirmed the classical laminated layer of hydatid cyst. Moreover, the ectocyst layer of the right lung showed the presence of numerous cluster septate hyphae with acute-angled branching, as seen in the morphology of *Aspergillus* species. DNA was extracted from the cyst, and the ITS1-5.8s-ITS2 region of the fungal agent was amplified. Sequencing and analysis of seminested PCR product revealed that the isolate has the most similarity with *Aspergillus niger*. Further attention is recommended to control fungal pathogens during pulmonary hydatidosis. The coexistence of aspergilloma should always be kept in mind for the better management of CE.

## 1. Background

Cystic echinococcosis (CE) is a zoonotic parasitic infection caused by the larval stage of *Echinococcus granulosus* [1]. The adult *E. granulosus* resides in the bowel of definitive host and releases eggs that are passed in the feces. These eggs are then ingested by a suitable intermediate host, including sheep, goat, swine, and cattle. Then, the eggs hatch in the bowels and release oncospheres, where they penetrate the intestinal wall. These oncospheres then migrate through the circulatory system to various organs of the host and develop into a hydatid cyst. Humans become infected through the ingestion of food contaminated by the worm eggs [1]. Although with humans as dead-end hosts any organ can be infected by this parasite, the liver and lung are the commonly involved organs. The disease is prevalent in many areas of the world including the Middle East in Asia [2, 3]. Coinfection of hydatid cyst with bacterial infections, e.g., *Haemophilus influenzae*, or viral infections has been previously reported [4, 5]. Occasionally, hydatid cyst might become infected with fungi including *Aspergillus* species [6–8]. *Aspergillus* species are saprophytic fungi with high potential to easily colonize within pulmonary cavities. Occasionally, aspergilloma reported as coexistent infection with hydatid disease might be seen in immunosuppressed or even immunocompetent CE patients [6, 7, 9, 10]. It needs to be mentioned that *Aspergillus* can cause hemoptysis, especially in immunosuppressed patients [6, 7].

Here, we reported an unusual coexistence of pulmonary hydatid cyst and aspergilloma or “fungus ball” in an immunocompetent patient.

## 2. Case Report

A 42-year-old Iranian healthy male farmer and smoker was admitted to the Department of Pulmonary and Critical Care at Kowsar Hospital in Shiraz, Fars Province (Southern Iran). The patient had a history of contact with domestic animals while living in the village. He did not have any past significant medical history to suggest immunosuppression. His physical examination showed normality with vital signs as a pulse rate of 98 beats per minute, respiratory rate of 28 breaths per minute, oxygen saturation of 98%, and blood pressure of 110/80 mmHg. Full laboratory blood analysis revealed a hemoglobin level of 12.8 g/dl, and there was no evidence of eosinophilia on peripheral blood examination. Serum electrolytes and liver function test results were within the normal ranges. He was nonreactive for HBs and HIV antigens.

**Figure 1:**
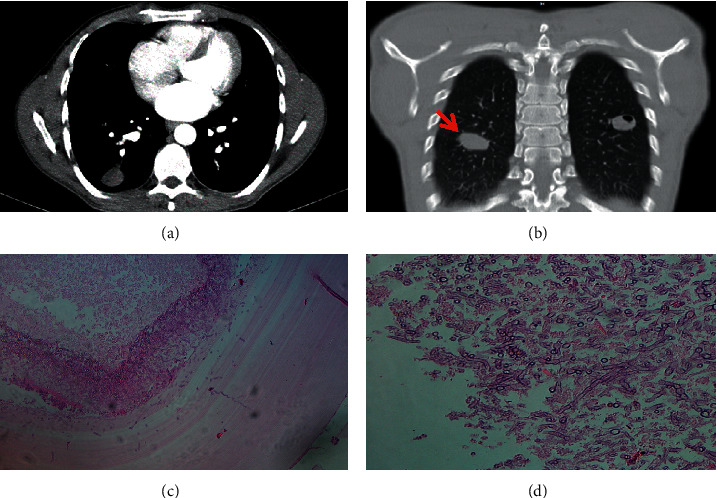
(a) CT scan (transverse view) revealing hydatid cyst in superior and inferior segment of the lower lobe of the right and left lungs, respectively. (b) CT scan (frontal view) indicates hydatid cyst in superior and inferior segment of the lower lobe of the right and left lungs, respectively. (c) Fungal hyphae on the outer chitinous wall of the hydatid cyst in the right lung (H&E ×100). (d) Photomicrograph showing groups of branching septate hyphae conforming to morphology of *Aspergillus* in hydatid cyst tissue in the right lung (H&E ×400).

The chest CT scan showed a restricted cystic lesion in the superior and inferior segment of the lower lobe of right and left lungs measuring about 27 mm and 22 mm, respectively (Figures 1(a) and 1(b)).

Ultrasound abdomen did not show any cysts in the liver or any other organs. Radical surgery (lobectomy) was performed, and the excised cysts were sent for histopathological examination. Gross examination of the sample showed a well-defined multilayered creamy-white cyst measuring 2.5 × 2 × 2 cm. Surprisingly, the ectocyst in the right lung showed the presence of numerous clusters of *Aspergillus* septate hyphae with acute-angled branching in hematoxylin and eosin stain (H&E) (Figures 1(c) and 1(d)). This finding was further confirmed by the periodic acid-Schiff (PAS) staining of the tissue section. Therefore, the diagnosis of dual infection by *Echinococcus* and *Aspergillus* was established. Moreover, the surrounding lung parenchyma around the cyst in the right lung showed acute and chronic inflammation and giant cell formation. Parietal pleura partial resection showed mild chronic inflammation. As the patient was immunocompetent, the postoperative course was uneventful and there was no dissemination of fungal or parasitic infection. The patient was discharged ten days postoperation and regularly followed up.

## 3. Molecular Evaluation

**Figure 2:**
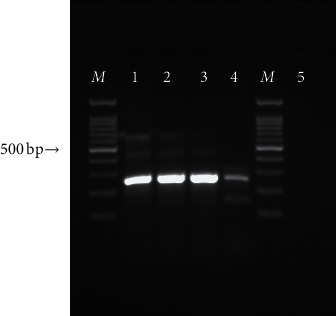
Agarose gel electrophoresis of the seminested PCR products of the ITS1-5.8s-ITS2 region. Line M, 100 bp molecular marker; lines 1 and 2, positive control (*Aspergillus niger)*; line 3, sample isolated from the patient in the current study; line 4, 1/10 diluted patient sample (*Aspergillus niger*); line 5, negative control.

Molecular evaluation of the tissue sample was performed for the identification of fungal agents. In brief, DNA was extracted from the paraffin-embedded tissue section, using the GeneAll kit (PCR Lab, Germany) with an additional bead-beating step to optimize fungal cell lysis. Elution buffer was used in the last step of DNA extraction, and the extracted DNA was stored at −20°C until use. A seminested PCR amplification was performed, targeting the ITS1-5.8s-ITS2 region and using the ITS1 (5-TCCGTAGGTGAACCTGCGG-3), ITS2 (5-GCTGCGTTCTTCATCGATGC-3), and ITS4 (5′TCCTCCGCTTATTGATATGC3′) primers [11, 12]. The PCR cycling conditions consisted of an initial denaturation step at 95°C for 5 minutes, followed by 38 cycles of denaturation at 95°C for 45 seconds, annealing at 58°C for 45 seconds, and extension at 72°C for 60 seconds. The PCR program was completed by a final extension at 72°C for 5 minutes. In the second step, amplification was performed using ITS1 and ITS2 primers at the same PCR condition. The amplified products were visualized, using 1.5% agarose gel containing 0.5 *μ*g/ml ethidium bromide stain (Figure 2). Sequencing was performed using the same primers as for the PCR.

The sequence of the isolate determined in this study was deposited in the GenBank database. The comparative DNA sequence analysis by nucleotide Basic Local Alignment Search Tool (BLAST) revealed that the amplified sequence was identified as *A. niger* with GenBank accession no. MT609916.1.

## 4. Discussion

The CE infection most commonly involves two blood filtering organs, the lungs and liver [13]. Pulmonary hydatidosis has been reported in association with cryptococcosis, aspergilloma, and saprophytic fungus [14, 15]. Aspergilloma is a saprophytic fungal infection that may cause broad-spectrum infections. Aspergilloma generally develops in the lung cavity including pre-existing cavities caused by several infections, e.g., tuberculosis, bronchiectasis, sarcoidosis, malignancies, and sometimes pulmonary infarcts. Coexistence of pulmonary hydatid cyst with fungi such as *Aspergillus* is extremely rare and has been recognized in only few cases [6–8, 10]. Aspergilloma in the hydatid cyst may be reported after the onset of surgery or many years later, whereas in the current case, the patient was involved with simultaneous aspergilloma and CE coinfection [6, 16]. In a retrospective analysis of 100 consecutive cases of hydatid cysts in Turkey, colonization by *Aspergillus* spp. was seen in two cases in the lung of immunocompetent patients [8]. Depending on the size and location of the hydatid cysts, the infection may remain asymptomatic for many years. Fungal colonization is usually followed by prior intervention or rupture of the cysts [17]. Although immunosuppressed patients are severely prone to aspergilloma, the coexistence of aspergilloma and hydatid cyst has been reported in both immunosuppressed and immunocompetent patients [18]. The presence of necrosis, granulomas with an unbalanced number of giant cells, and a neutrophilic infiltrate is a morphological alert to search for the aspergilloma agent. Early diagnosis and proper treatment are vital to prevent probable complications, such as massive hemoptysis or even invasive disease, resulted from infection by these two pathogens. The patient was prescribed 10 mg/kg/day of oral mebendazole for 3 months. However, 52% of all coinfected cases have been treated with azole agents, i.e., itraconazole, as reported by recent studies [7, 17]. Nevertheless, antifungal therapy has been recommended for three months postoperation, especially in immunosuppressed patients who might be at risk of additional *Aspergillus* infection.

## 5. Conclusion

We reported a case of aspergilloma in a patient with CE by histopathological and molecular analysis. Further attention is recommended to control fungal pathogens during pulmonary hydatidosis. The coexistence of aspergilloma should always be kept in mind for the better management of CE. This coinfection may be life-threatening, especially in immunosuppressed patients, and follow-up and prophylactic chemotherapy for aspergillosis may be useful to prevent further complications.

## Data Availability

The data used to support the findings of this study were supplied by Shiraz University of Medical Science under license and so cannot be made freely available. Requests for access to these data should be made to Kamiar Zomorodian, zomorodian@sums.ac.ir.
